# Comorbid Anxiety and Suicidal Behaviors in American Adolescents With Major Depression

**DOI:** 10.7759/cureus.8598

**Published:** 2020-06-13

**Authors:** Keerthika Mathialagan, Ozge Ceren Amuk, Noha Eskander, Rikinkumar S Patel

**Affiliations:** 1 Psychiatry, Sree Balaji Medical College and Hospital, Chennai, IND; 2 Psychiatry, Koç University School of Medicine, Istanbul, TUR; 3 Psychiatry, California Institute of Behavioral Neurosciences & Psychology, Fairfield, USA; 4 Psychiatry, Griffin Memorial Hospital, Norman, USA

**Keywords:** major depressive disorder, mdd, suicide and depression, suicidal behavior, childhood anxiety disorders, comorbid anxiety, child and adolescent psychiatry, child and adolescent

## Abstract

Objective

The aim of this study was to evaluate the odds of association between suicidal behaviors and comorbid anxiety disorders in adolescents with major depressive disorder (MDD).

Methods

We included 122,020 adolescent inpatients with MDD from the Nationwide Inpatient Sample (NIS) and further grouped them by co-diagnosis of anxiety disorders. Logistic regression analysis was used to evaluate the odds ratio (OR) of suicidal behaviors due to comorbid anxiety disorders.

Results

Out of total MDD inpatients, 45.8% had comorbid anxiety disorders. Around 53.5% MDD inpatients with anxiety disorders had suicidal behaviors, which were significantly higher than seen in 52.6% non-anxiety cohort (P = 0.002). Comorbid anxiety disorders had a minimally positive association with suicidal behaviors and were not statistically significant (OR: 1.01; P = 0.710) after controlling the logistic regression analysis for demographic confounders and psychiatric comorbidities. MDD inpatients with comorbid psychotic disorders were positively associated (OR: 1.16; P = 0.007) with suicidal behaviors.

Conclusions

MDD with comorbid anxiety had a statistically non-significant association with suicidal behaviors in adolescents. Depression has a direct and independent effect on adolescent suicidal behaviors, whereas anxiety has a direct effect only on perpetuating depression. Early diagnosis and management of comorbid anxiety and psychosis with MDD reduce functional impairment and suicide risk in at-risk populations.

## Introduction

Major depressive disorder (MDD) in adolescents is a serious and potentially fatal problem. Earlier age of onset is associated with a prolonged and severe course and greater illness burden [1]. Over the past 25 years, the age of onset of MDD appears to have consistently decreased, and several etiologies have been implicated in early-onset MDD including genetic, familial, and environmental factors [1,2]. By 2017, an estimated 3.2 million (13.3%) adolescents aged 12 to 17 years in the United States had at least one major depressive episode [3]. The prevalence of MDD was higher among adolescent females (20%) when compared with adolescent males (6.8%) and was highest among adolescents reporting two or more races (16.9%) [3]. A confluence of hormonal and neurodevelopmental changes that vary by sex during the pubertal transition seems to influence the gender difference in depression [4].

The total economic burden of MDD is now estimated to be $210.5 billion annually. Although nearly half (48%-50%) of these costs are attributed to the workplace, 45% to 47% is due to direct medical costs (outpatient and inpatient medical services, pharmacy), and around 5% ($9.7 billion) of the total expenditures are related to suicides [5].

It is imperative to identify adolescents at risk of suicide due to MDD since suicidal ideation is associated with earlier age of onset, longer depressive episodes, and earlier relapse of depression [1]. The major predictor for suicide among adolescents is the previous history of a suicide attempt. Other major risk factors are anxiety disorders, eating disorders, alcohol and/or drug abuse, impulsive aggression, antisocial behavior, family history of suicidal behavior, and family conflict and discordance [6]. Also, two nationwide studies conducted in the United States found a significant association between suicidality and post-traumatic stress disorder, with attention-deficit/hyperactivity disorder (ADHD) increasing suicide risk in depressed adolescents by 52% [7,8].

Individuals with mood disorders are also at a higher risk of association with comorbid anxiety disorders, including panic disorder, obsessive-compulsive disorder (OCD), and social anxiety disorder. Likewise, those with anxiety disorders are at a higher risk of lifetime or concurrent mood disorder [9]. Anxiety disorders are the most common psychiatric illness in children and adolescents, with prevalence rates of 5.7% to 17.7% [10-12]. A significant proportion of these children with anxiety disorders experience chronic courses of the disease lasting through adulthood and these individuals are at a higher risk of developing depressive disorders [13,14].

The likelihood of suicide is increased in depression with comorbid anxiety relative to the risk of suicide with either depression or anxiety alone [14,15]. Our study focused firstly to understand the demographics and psychiatric comorbidities in adolescents with MDD and comorbid anxiety disorders. The second goal is to assess the odds of association between suicidal behaviors and comorbid anxiety disorders in adolescents with MDD.

## Materials and methods

Data source

We conducted a cross-sectional analysis using the National Inpatient Sample (NIS) from January 2012 to December 2014 [16]. The NIS data include inpatient data from 4,400 non-federal hospitals across 44 states in the United States. The primary and co-diagnostic information is identified using the International Classification of Diseases, Ninth Revision (ICD-9), and Clinical Classification Software (CCS) codes [16]. The NIS is a de-identified dataset and therefore this study does not require approval from the Institutional Review Board [16].

Inclusion criteria and outcome variables

We included 122,020 adolescent inpatients (age 12-17 years) with a primary diagnosis of MDD using the following ICD-9 codes: 296.20-296.26 or 296.30-296.36. We then compared the groups with versus without comorbid diagnosis of anxiety disorders using the CCS code 651 (N = 55,840) versus without comorbid anxiety disorders (N = 66,180). The co-diagnosis of suicide and intentional self-inflicted injury was detected using CCS code 662.

Demographic characteristics studied were age, sex (male or female), and race (white, black, Hispanic, and native Americans (NA)/Asians) [17]. The comorbidities included in our study are ADHD/conduct/behavioral disorders (CCS code 652), psychotic disorders (CCS code 659), alcohol abuse (CCS code 660), and substance abuse (CCS code 661) [17].

Statistical analysis

We used cross-tabulation model and Pearson’s chi-square test to measure the differences in demographic and psychiatric comorbidities, and suicidal behavior in MDD inpatients with anxiety vs. non-anxiety cohorts. Logistic regression analysis was used to evaluate the odds ratio (OR) of suicidal behaviors in inpatients with versus without comorbid anxiety after controlling for demographic confounders and psychiatric comorbidities. A P-value of <0.01 was considered for statistical significance, and all the analyses were conducted using SPSS Version 26 (IBM Corp., Armonk, NY, USA).

## Results

Our sample population of 122,020 adolescent MDD inpatients were majorly females (73.1%) and whites (65.4%). Out of total MDD inpatients, 45.8% had comorbid anxiety disorders. A higher proportion of these inpatients with anxiety were females (77.4%) compared with 69.4% of females in the non-anxiety cohort. Also, MDD with anxiety was prevalent in whites (71%) followed by Hispanics (12%) and blacks (9.2%).

Around 53.5% MDD inpatients with anxiety had suicidal behaviors, which was significantly higher than that seen in 52.6% non-anxiety cohort (P = 0.002). The most prevalent psychiatric comorbidities in MDD inpatients with anxiety were ADHD/conduct disorder/disruptive behavioral disorders (24.2%) and substance abuse (15.3%), but there was statistically non-significant difference when compared with the non-anxiety cohort, as shown in Table 1.

**Table 1:** Demographic and comorbidities in major depressive disorder inpatients NA, native American; ADHD, attention-deficit/hyperactivity disorder

Variable	Comorbid anxiety disorders		P-value
	(-) in %	(+) in %	
Total inpatients	66,180	55,840	-
Mean age, years	14.83	14.92	<0.001
Sex			
Male	30.6	22.6	<0.001
Female	69.4	77.4	
Race			
White	61.0	71.0	<0.001
Black	12.6	9.2	
Hispanic	17.3	12.0	
NA/Asians	9.1	7.8	
Comorbidities			
ADHD/conduct/behavioral disorder	24.2	24.2	0.959
Psychotic disorders	1.1	1.5	<0.001
Suicidal behaviors	52.6	53.5	0.002
Alcohol abuse	5.0	5.8	<0.001
Substance abuse	15.3	15.3	0.780

There existed a statistically non-significant association between age and sex with suicidal behaviors in MDD inpatients. When compared with whites, other races had a negative association with suicidal behaviors, and the result was statistically significant. Comorbid anxiety disorder had a minimally positive association with suicidal behaviors and was not statistically significant (OR: 1.01; 95% CI: 0.98-1.03; P = 0.710). The comorbid psychotic disorder was positively associated with suicidal behavior in MDD inpatients (OR: 1.16; 95% CI: 1.04-1.29; P = 0.007). Alcohol abuse and substance abuse relationship with suicidal behaviors in MDD inpatients was not statistically significant, as shown in Table 2.

**Table 2:** Predictors of suicidal behaviors in major depressive disorder inpatients OR, odds ratio; CI, confidence interval; NA, native American; ADHD, attention-deficit/hyperactivity disorder

Variable	Logistic regression analysis		
	OR	95% CI	P-value
Age	1.0	0.99-1.01	0.936
Sex			
Male	Reference		
Female	1.02	0.99-1.05	0.210
Race			
White	Reference		
Black	0.95	0.92-0.99	0.016
Hispanic	0.89	0.86-0.92	<0.001
NA/Asians	0.84	0.81-0.88	<0.001
Comorbid anxiety disorder			
No	Reference		
Yes	1.01	0.98-1.03	0.710
Comorbidities			
ADHD/conduct/behavioral disorder	0.89	0.87-0.92	<0.001
Psychotic disorders	1.16	1.04-1.29	0.007
Alcohol abuse	1.02	0.96-1.08	0.573
Substance abuse	0.98	0.94-1.02	0.272

## Discussion

We found that MDD is more commonly seen in females (73.1%), which could be due to a matrix of social, behavioral, psychological, and biological factors. Women are at a higher risk of anxiety during childhood and adolescence, find themselves either limited or burned out in their sociocultural role, and are more sensitive with poor coping skills to adverse events in life and depression [18]. Although genetics has minimal effect on gender differences, genetic factors could indirectly increase vulnerability to depression in females [18]. Around three-fourths of depressed adolescents with comorbid anxiety seen in our study were females. MDD with comorbid anxiety disorders was found to be higher among female patients who were single with poor physical and psychological quality of life and poor support systems [19]. Although there was a female preponderance in regard to comorbid disorder, there were no significant differences related to family income and education anxiety [19].

Mood disorders are associated with several comorbid conditions, with the most prevalent comorbidity being anxiety disorders. Around half of the adolescents with MDD had comorbid anxiety in our study, which correlates with a face-to-face household survey in 9,090 adults [20]. The prevalence of depression with comorbid anxiety was found predominantly in white adolescents, followed by Hispanics and African Americans. Exposure to significant stressors in life leads to anxiety, which, in turn, facilitates further decompensation, leading to major depression [21]. Patients with depressive and anxiety disorders share similar exposure and rumination to stressful life events that lead to subsequent increases in both anxiety and depression [22].

Depressed children and adolescents with comorbid anxiety are at a higher risk of alcohol and substance abuse, with around 75% of current substance use disorders meeting the criteria for mood and anxiety disorders [23]. In our study, we focused on adolescents (12-18 years) with MDD only and found no statistically non-significant difference in the prevalence of substance abuse by the presence of comorbid anxiety disorders. Adolescents with MDD are at a higher risk of suicidal ideations, with a prevalence rate of 49% to 64% [24]. Suicide attempts among adolescents range from 1.3% to 3.8% in males and from 1.5% to 10.1% in females [25]. Few studies in the adult population have found increased rates of suicidal ideation, suicide attempts, and completed suicide in patients with comorbid anxiety and depression compared with those with a single psychiatric illness [15,26].

In a longitudinal epidemiological study including children aged 9 to 16 years, the risk of suicide was higher in depressed children with comorbid anxiety and disruptive disorders, but pure anxiety disorders did not result in increased suicidality [27]. Although the severity of psychiatric impairment was an independent risk factor for increased suicidality, children with depression and generalized anxiety disorder (GAD) were still at a higher risk of suicide after controlling for severity impairment [27]. The features of GAD may interact with features of depression, perpetuating the risk of suicidality [27]. A large, prospective study conducted as a part of the program of the Oregon Adolescent Depression Project found that the rates of suicide attempt did not significantly differ between those with comorbid anxiety-MDD and pure MDD (without comorbid anxiety) [28]. A history of anxiety disorder only predicted a future episode of depression, whereas depression predicted future suicide attempts and further depressive episodes [28]. The study found that unlike pure depression, suicide attempts rates in pure anxiety disorders were relatively low, and the difference in rates of suicide attempts between pure MDD and MDD with comorbid anxiety was not statistically significant [28]. These study findings are consistent with our data analysis results as comorbid anxiety disorder had a statistically non-significant and small positive association with suicidal behaviors after controlling the logistic regression model for demographic confounders and psychiatric comorbidities.

Psychotic disorders were positively associated (16% increase) with suicidal behaviors in adolescents with MDD. Our finding was supported by a study by Fredriksen et al. that found that patients with psychotic major depression (PMD) are at a 1.2-fold higher lifetime risk of committing suicide [29]. Patients with PMD during an MDD episode experience psychotic phenomena, are more impulsive, have intense feelings of guilt and anxiety, and, being in their disorganized mental state, are unable to control their actions, which lead to increased suicidal behavior [29]. In a meta-analysis study, patients with PMD had an elevated risk of suicide compared with severely depressed patients with PMD [30].

Our study has some limitations. The study is based on data collected from the NIS and lacks patient-level clinical information. The prevalence of comorbidities in study participants may differ when compared with the general population as our participants were chosen from an inpatient sample. Also, our study sample largely consists of whites, and results might not truly represent people from other races or ethnicity. One of the strengths of the study is that the NIS has the capacity to build population-based inpatient representation of associations between diseases and comorbidities. The chances of recall bias are minimal given that the NIS has primary and secondary diagnostic codes and other clinical information obtained at the time of hospitalization. Another strength of this study is in its large sample size of 122,020 inpatients and data reliability, as the information is coded independently of the individual practitioner; this would, therefore, minimize reporting bias, and the large sample size increases the power to detect differences.

## Conclusions

MDD with comorbid anxiety had a statistically non-significant association with suicidal behaviors in adolescents. Depression has a direct and independent effect on adolescent suicidal behaviors, whereas anxiety has a direct effect only on perpetuating depression. Depressed adolescents with comorbid psychosis were associated with an increased risk of suicidal behaviors by 16%. Early diagnosis and management of comorbid anxiety and psychosis with MDD reduce functional impairment and suicides in at-risk populations.

## References

[1] Greden JF (2001). The burden of recurrent depression: causes, consequences, and future prospects. J Clin Psychiatry.

[2] Jaffee SR, Moffitt TE, Caspi A, Fombonne E, Poulton R, Martin J (2002). Differences in early childhood risk factors for juvenile-onset and adult-onset depression. Arch Gen Psychiatry.

[3] (2020). Major depression. https://www.nimh.nih.gov/health/statistics/major-depression.shtml.

[4] Salk RH, Hyde JS, Abramson LY (2017). Gender differences in depression in representative national samples: meta-analyses of diagnoses and symptoms. Psychol Bull.

[5] Greenberg PE, Fournier AA, Sisitsky T, Pike CT, Kessler RC (2015). The economic burden of adults with major depressive disorder in the United States (2005 and 2010). J Clin Psychiatry.

[6] Vitiello B, Pearson JL (2008). A depressed adolescent at high risk of suicidal behavior. Am J Psychiatry.

[7] Eskander N, Vadukapuram R, Zahid S, Ashraf S, Patel RS (2020). Post-traumatic stress disorder and suicidal behaviors in American adolescents: analysis of 159,500 psychiatric hospitalizations. Cureus.

[8] Zahid S, Bodicherla K, Eskander N, Patel RS (2020). Attention-deficit/hyperactivity disorder and suicidal risk in major depression: analysis of 141,530 adolescent hospitalizations. Cureus.

[9] Pine DS, Cohen P, Gurley D, Brook J, Ma Y (1998). The risk for early-adulthood anxiety and depressive disorders in adolescents with anxiety and depressive disorders. Arch Gen Psychiatry.

[10] Bernstein GA, Borchardt CM, Perwien AR (1996). Anxiety disorders in children and adolescents: a review of the past 10 years. J Am Acad Child Adolesc Psychiatry.

[11] Cohen P, Cohen J, Kasen S (1993). An epidemiological study of disorders in late childhood and adolescence--I. Age- and gender-specific prevalence. J Child Psychol Psychiatry.

[12] (2020). Prevention of mental disorders: effective interventions and policy options. http://www.who.int/mental_health/evidence/en/prevention_of_mental_disorders_sr.pdf.

[13] Beesdo-Baum K, Knappe S (2012). Developmental epidemiology of anxiety disorders. Child Adolesc Psychiatr Clin N Am.

[14] Melton TH, Croarkin PE, Strawn JR, McClintock SM (2016). Comorbid anxiety and depressive symptoms in children and adolescents: a systematic review and analysis. J Psychiatr Pract.

[15] Mykletun A, Bjerkeset O, Dewey M, Prince M, Overland S, Stewart R (2007). Anxiety, depression, and cause-specific mortality: the HUNT study. Psychosom Med.

[16] (2020). Overview of the National (Nationwide) Inpatient Sample (NIS). https://www.hcup-us.ahrq.gov/nisoverview.jsp.

[17] (2020). NIS description of data elements. https://www.hcup-us.ahrq.gov/db/nation/nis/nisdde.jsp.

[18] Picco L, Subramaniam M, Abdin E, Vaingankar JA, Chong SA (2017). Gender differences in major depressive disorder: findings from the Singapore mental health study. Singapore Med J.

[19] Zhou Y, Cao Z, Yang M (2017). Comorbid generalized anxiety disorder and its association with quality of life in patients with major depressive disorder. Sci Rep.

[20] Kessler RC, Berglund P, Demler O (2003). The epidemiology of major depressive disorder: results from the National Comorbidity Survey Replication (NCS-R). JAMA.

[21] Hirschfeld RM (2001). The comorbidity of major depression and anxiety disorders: recognition and management in primary care. Prim Care Companion J Clin Psychiatry.

[22] Michl LC, McLaughlin KA, Shepherd K, Nolen-Hoeksema S (2013). Rumination as a mechanism linking stressful life events to symptoms of depression and anxiety: longitudinal evidence in early adolescents and adults. J Abnorm Psychol.

[23] Kandel DB, Johnson JG, Bird HR (1999). Psychiatric comorbidity among adolescents with substance use disorders: findings from the MECA study. J Am Acad Child Adolesc Psychiatry.

[24] Gould MS, Greenberg T, Velting DM, Shaffer D (2003). Youth suicide risk and preventive interventions: a review of the past 10 years. J Am Acad Child Adolesc Psychiatry.

[25] Bridge JA, Goldstein TR, Brent DA (2006). Adolescent suicide and suicidal behavior. J Child Psychol Psychiatry.

[26] Sareen J, Cox BJ, Afifi TO, de Graaf R, Asmundson GJ, ten Have M, Stein MB (2005). Anxiety disorders and risk for suicidal ideation and suicide attempts: a population-based longitudinal study of adults. Arch Gen Psychiatry.

[27] Foley DL, Goldston DB, Costello EJ, Angold A (2006). Proximal psychiatric risk factors for suicidality in youth: the Great Smoky Mountains Study. Arch Gen Psychiatry.

[28] Lewinsohn PM, Rohde P, Seeley JR (1996). Adolescent suicidal ideation and attempts: prevalence, risk factors, and clinical implications. Clin Psychol.

[29] Fredriksen KJ, Schoeyen HK, Johannessen JO, Walby FA, Davidson L, Schaufel MA (2017). Psychotic depression and suicidal behavior. Psychiatry.

[30] Gournellis R, Tournikioti K, Touloumi G (2018). Psychotic (delusional) depression and completed suicide: a systematic review and meta-analysis. Ann Gen Psychiatry.

